# Complete genome sequence of new bacteriophage phiE142, which causes simultaneously lysis of multidrug-resistant *Escherichia coli* O157:H7 and *Salmonella enterica*

**DOI:** 10.1186/s40793-016-0211-5

**Published:** 2016-12-13

**Authors:** Luis Amarillas, Cristobal Chaidez, Arturo González-Robles, Josefina León-Félix

**Affiliations:** 1 Laboratorio de Biología Molecular y Genómica Funcional, Centro de Investigación en Alimentación y Desarrollo, A.C, Culiacán, Sinaloa Mexico; 2 Laboratorio de Genética, Instituto de Investigación Lightbourn. A. C, Cd. Jiménez, Chihuahua Mexico; 3 Laboratorio Nacional para la Investigación en Inocuidad Alimentaria, Centro de Investigación en Alimentación y Desarrollo, Culiacán, Sinaloa Mexico; 4 Departamento de Infectómica y Patogénesis Molecular, Centro de Investigación y de Estudios Avanzados, Instituto Politécnico Nacional, Ciudad de México, Mexico

**Keywords:** Short genome report, phiE142, *Enterobacteriaceae* bacteriophage, Genome sequence, Potential biocontrol agent

## Abstract

The emergence of antibiotic-resistant foodborne bacteria is a global health problem that requires immediate attention. Bacteriophages are a promising biotechnological alternative approach against bacterial pathogens. However, a detailed analysis of phage genomes is essential to assess the safety of the phages prior to their use as biocontrol agents. Therefore, here we report the complete genome sequence of bacteriophage phiE142, which is able to lyse *Salmonella* and multidrug-resistant *Escherichia coli* O157:H7 strains. Bacteriophage phiE142 belongs to the *Myoviridae* family due to the presence of long non-flexible tail and icosahedral head. The genome is composed of 121,442 bp and contains 194 ORFs, and 2 tRNAs. Furthermore, the phiE142 genome does not contain any genes coding for food-borne allergens, antibiotics resistance, virulence factors, or associated with lysogenic conversion. The bacteriophage phiE142 is characterized by broad host range and compelling genetic attributes making them potential candidates as a biocontrol agent.

**Electronic supplementary material:**

The online version of this article (doi:10.1186/s40793-016-0211-5) contains supplementary material, which is available to authorized users.

## Introduction

Foodborne diseases are an important cause of morbidity and mortality worldwide, therefore are a serious public health problem [1]. Bacteria cause the majorities of foodborne illnesses; *Escherichia coli* and *Salmonella* are among the most common foodborne pathogens that affect millions of people annually [2]. Furthermore, the emergence of antimicrobial resistance *E. coli* and *Salmonella* strains makes more difficult its control [3]. Hence, novel control methods for reducing the risk of bacterial food contamination, which are both environmental friendly, are urgently needed.

In this context, bacteriophages have several potential applications in the food industry; these killing-bacteria viruses are alternatives to conventional antimicrobials method for the control of pathogenic bacteria and have great potential in the improvement of food safety [4–6]. Bacteriophages suitable for biocontrol purposes must be genetically sequenced to ensure that are strictly lytic (always lyse infected cells host), does not encode any bacterial virulence factors or proteins with a potential to cause allergenicity [7, 8].

The primary aim of our research group is increase knowledge of phage biodiversity and contribute to the understanding of different types of phages in several regions of Sinaloa, an important agricultural region in Northwestern Mexico. Recently, a new bacteriophage, designated as phiE142, one of phages isolated, exhibits a high potential as a biocontrol agent [9]. However, information about genome of phage phiE142 is still limited; therefore, to further understand the phage biology, the genome was sequenced.

## Organism information

### Classification and features

The bacteriophage phiE142 was previously isolated in Food and Environmental Microbiology Laboratory at the Research Center for Food and Development from animal feces samples collected on a farm in Northwestern Mexico. An *E. coli* strain EC-48 (bacterial used for bacteriophage propagation and titration), was also isolated from the same geographical region two years before the isolation of the phage [10]. Phage phiE142 produced clear plaques of 2 to 3 mm in diameter on the *E. coli* EC-48 lawn; the plaques were already visible after four to six hours of incubation time at 37 °C.

We analyzed the lytic host range of phage using spot tests assays of different bacterial, including 48 *Salmonella* strains and 33 *E. coli* strains (Additional file 1: Table S1). Based upon spot testing results, the phage phiE142 had lytic activity against 76% of the *E. coli* strains and 29% of *Salmonella* strains tested. These results indicate that bacteriophage phiE142 has the potential to be evaluated as an alternative strategy to biocontrol of *E. coli* and *Salmonella*.

**Fig. 1:**
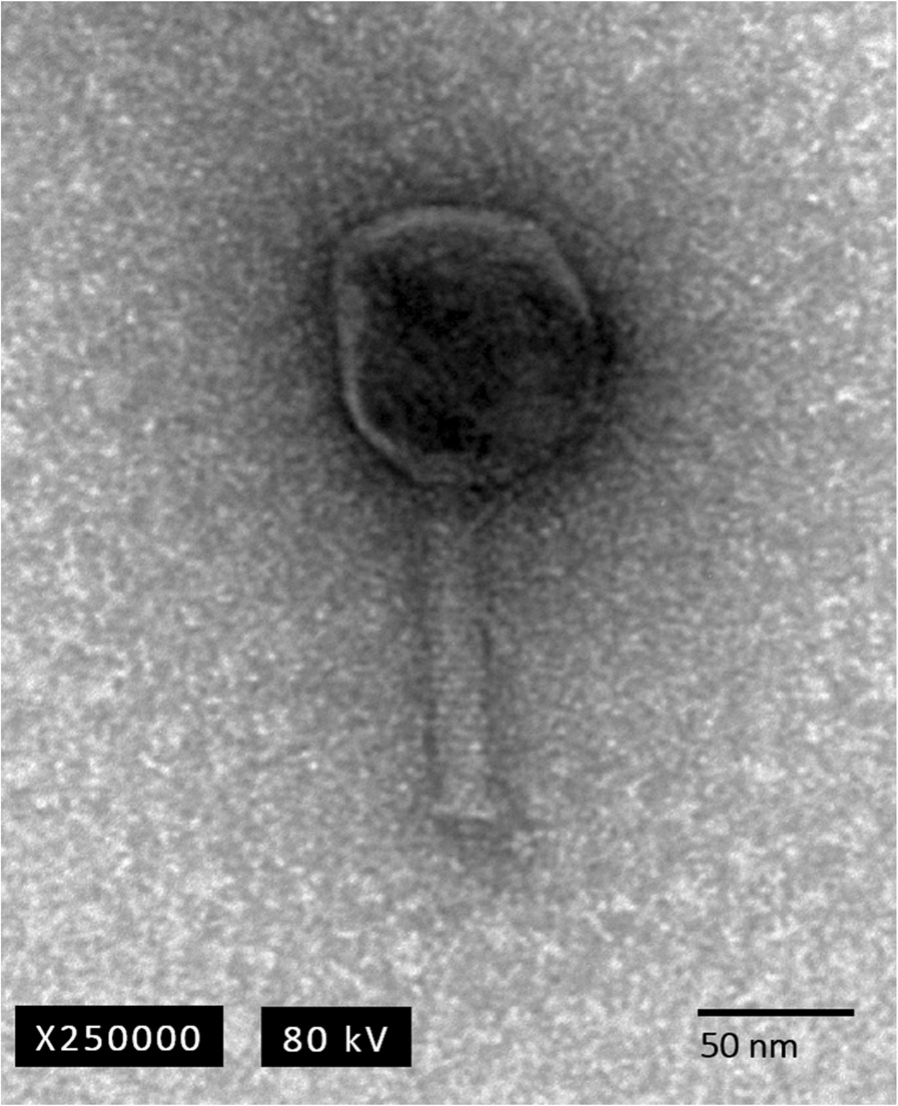
Transmission electron microscopy (TEM) of bacteriophage phiE142, which exhibits an icosahedral head, long and non-flexible tail. This morphology corresponds to the *Myoviridae* family

The phiE142 phage was stained with 2% uranyl acetate and examined by transmission electron microscopy (TEM) and classified into its appropriate viral morphotype according to Ackermann’s classification [11]. The analysis suggests that phage phiE142 belongs to the order *Caudovirales* and family *Myoviridae* based on the presence of almost isometric head with an average diameter of ∼ 58 nm, long non-flexible contractile tail about 120 nm in length (Fig. 1) [12]. Phage phiE142 has a genome of 121,442 bp, with a coding region of 94.4%, GC content of 37.4%, and the gene density is 1.60. It contains 194 coding sequences ranging from 102 bp to 3,300 bp, with 53 genes on the positive strand and 141 genes on the negative strand. Phylogenetic characteristics of this phage are indicated in Table 1.

**Table 1:** Classification and general features of Enterobacteria phage phiE142 according to the MIGS recommendation [29]

MIGS ID	Property	Term	Evidence code^a^
	Classification	Domain: viruses, dsDNA viruses, no RNA stage	TAS [11]
		Phylum: unassigned	
		Class: unassigned	
		Order: *Caudovirales*	TAS [11]
		Family: *Myoviridae*	TAS [11]
		Genus: unassigned	
		Species: unassigned	
		Strain: phiE142	
	Gram stain	Not-applicate	
	Particle shape	Icosahedral head with long contractile tail	IDA
	Motility	Not-applicate	IDA
	Sporulation	Not-applicate	IDA
	Temperature range	Not-reported	
	Optimum temperature	Not-reported	
	pH range; Optimum	Not-reported	
	Carbon source	Not-applicate	
MIGS-6	Habitat	Equine gut	IDA
MIGS-6.3	Salinity	Not-reported	
MIGS-22	Oxygen requirement	Not-applicate	
MIGS-15	Biotic relationship	Intracellular parasite of *E. coli* strain EC-48	IDA
MIGS-14	Pathogenicity	Lytic phage of *E. coli* strain EC-48	IDA
MIGS-4	Geographic location	Elota, Sinaloa, México	IDA
MIGS-5	Sample collection	March 04, 2014	IDA
MIGS-4.1	Latitude	23°54′35.8″N	IDA
MIGS-4.2	Longitude	106°54′28.2″W	IDA
MIGS-4.3	Depth	0 m	IDA
MIGS-4.4	Altitude	20 m	IDA

^a^ Evidence codes - *IDA* Inferred from Direct Assay, *TAS* Traceable Author Statement. These evidence codes are from the Gene Ontology project [30]

**Fig. 2:**
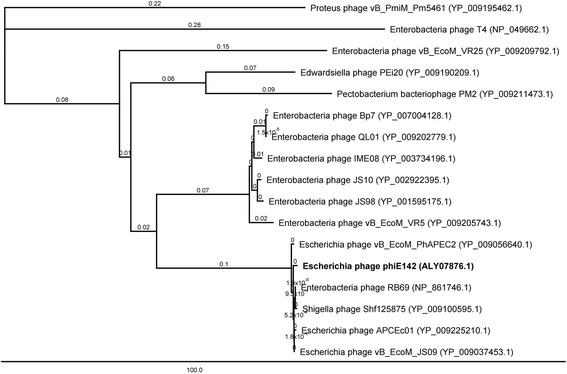
Phylogenetic tree based on the predicted amino acid sequences of the DNA polymerase of 17 bacteriophages. Phylogenetic tree was performed in MEGA 6.0 version by the neighbor joining method with the Jukes Cantor model. The percentages of bootstrap samples (of 1,000) are indicated for each internal branch. The scale bar indicates the proportion of substitutions per site

**Fig. 3:**
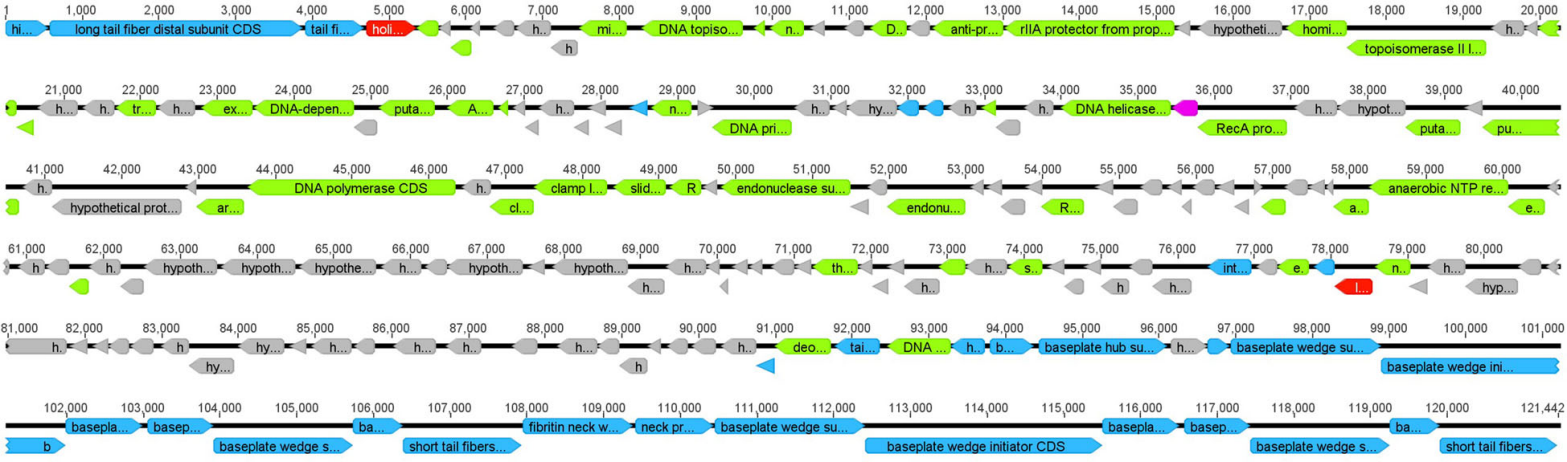
Diagram of bacteriophage genome. The arrows indicated open reading frame (ORF), the orientation of which shows the direction of transcription. Putative genes are colored according to the predicted functions of their products: Phage structural proteins (blue arrows), DNA regulation module (green arrows), packaging module (pink arrows), host lysis proteins (red arrows), and hypothetical proteins (gray arrows)

The sequence of DNA polymerase has become a commonly-used marker for constructing phylogenetic analysis, therefore the phylogenetic tree was performed based of DNA polymerase deduced amino acid sequences. According to the phylogenetic tree, the phage phiE142 and others eight phages that infect the bacterial family *Enterobacteriaceae* were clustered in the same group (Figs. 2 and 3). All of these phages are members of the *Tevenvirinae* subfamily and are strictly lytic (Based on PHACTS program server). Considering the close relationship among these phages, it is likely that phiE142 also belongs to this genus. This result confirms the findings obtained by electron microscopy.

## Genome sequencing information

### Genome project history

The bacteriophage phiE142 is one of the first genome to be completely sequenced publicly available for a phage infecting *E. coli* and *Salmonella* strains isolated from environmental sources in Northwest Mexico. The analysis of more genomes of bacteriophages is necessary to increase our understanding of the genetic diversity of bacteriophages, phage biology, basic molecular mechanisms, and provide a deeper insight into the relationship of phages with their hosts. Furthermore, analysis of phage genomes may reveal novel antimicrobial peptides and enzymes with bactericidal activity. In addition, the genome well understood is an essential requisite to ensure the safety of the phages prior to their use as biocontrol agents. Therefore, the genome project was deposited in the Genomes On Line Database (GOLD). The genome sequence of bacteriophage phiE142 was deposited in GenBank under accession number KU255730. The summary of genome project is available in the Table 2.

**Table 2:** Project information

MIGS ID	Property	Term
MIGS 31	Finishing quality	Finishing
MIGS-28	Libraries used	Standard Illumina paired-end
MIGS 29	Sequencing platforms	Illumina HiSeq
MIGS 31.2	Fold coverage	~10,000×
MIGS 30	Assemblers	Velvet-Geneious R8
MIGS 32	Gene calling method	Geneious R8
	Locus Tag	phiE142_
	Genbank ID	KU255730.1
	GenBank Date of Release	January 19, 2016
	GOLD ID	Gp0128385
	BIOPROJECT	NA^a^
MIGS 13	Source Material Identifier	NA^a^
	Project relevance	Bacteriophage candidate as a biological control agent

### Growth conditions and genomic DNA preparation

Standard double-layer agar plate method was used to obtain high-titer stocks of the phage phiE142 [13], with some modifications. Briefly, 100 μl of phage stock and 1 ml of overnight culture of *E. coli* strain EC-48 were mixed with 3 ml TSB with 0.4% agarose, spread on TSA plates, and incubated overnight at 37 °C. After, phage was subsequently collected by adding 6 ml of SM buffer (50 mM Tris-HCl, pH 7.5, 0.1 M NaCl, 8 mM MgSO_4_, 0.01% gelatin) to the surface of each plate and the soft agar was scraped off the surface of the agar plates. Cell debris was removed by subsequent centrifugation at 5,500 × *g* for 10 min, the supernatant was filtered with 0.22 μm syringe filters, and phage particles were precipitated by centrifugation at 40,000 × *g* at 4 ° C for 2 h. The phage pellet was suspended in SM buffer and stored at 4°C. Bacteriophage DNA was isolated by the method of proteinase K and phenol–chloroform as previously described [14], with minor modifications. One milliliter of purified phage suspension was treated with 1 μg/ml of DNaseI and RNaseA (Sigma-Aldrich) at 37 °C for 1 h. Subsequently, sodium dodecyl sulfate (final concentration, 0.5%), EDTA (20 mM, pH 8.0), and proteinase K (final concentration, 25 μg/ml) were added, and the suspension was incubated at 56 °C for 1 h. After proteins were removed by an equal volume of phenol-chloroform (1:1), and DNA was precipitated from the aqueous phase by cold ethanol. Following centrifugation at 15, 000 × *g* for 15 min at 4 °C, the pellet was washed twice with 70% ethanol, centrifuged at the same conditions. Finally, the dried DNA pellet was suspended in nuclease-free water. Concentration of phage DNA was estimated with a NanoDrop spectrophotometer (Thermo Fisher Scientific, Wilmington, DE) and also the quality of extracted DNA was also tested visually with electrophoresis on a 1% agarose.

### Genome sequencing and assembly

High-throughput DNA Sequencing of phage genomic DNA was performed using HiSeq 2000 technology (Illumina) to produce 100 bp paired-end reads, library construction and sequencing were performed according to the manufacturer’s instructions. In total, about 18 million pair reads of 100 bases in length were obtained with a quality filter threshold of Q30. The reads were analyzed and quality checked using FastQC and Geneious software package R8 (Biomatters Ltd., New Zealand) was used to trim raw reads with a low quality score. The *de novo* assembly was conducted with Velvet (implemented in Geneious, running VelvetOptimiser for selection of *k*-mer), resulting in one final contig with coverage from approximately 10,000-fold. Additional manual functional annotation and genome map was performed using Geneious software.

### Genome annotation

Open reading frames (ORFs) were identified using Glimmer 3.02 [15], GeneMark.hmm [16], and ORF Finder [17]. The putative functions of the ORFs were analyzed by protein BLASTp searches, with a cut off *E* value of 10^−4^. Predicted protein sequences were analyzed against InterProScan [18], Pfam [19] and TMHMM Server version 2.0 [20] for conservative domain identification. Signal peptides were predicted using SignalP 4.1. The search of putative tRNA encoding genes was done using ARAGORN [21] and tRNAscan-SE [22]. The origin of replication was predicted using a GC-skew plot generated by GenSkew [23]. Moreover, all identified ORFs were compared against the virulence factor database [24] and the ResFinder database [25]. Additionally, the predicted phage protein sequences were searched to identify proteins potentially allergenic using tools from the Food Allergy Research and Resource Programme [26]. The lifestyle of the phages was predicted using the PHACTS program [27]. Whole genome comparisons were carried out using Mauve [28].

## Genome properties

The detailed annotation information for phage genome was summarized in Table 3. The phage has a DNA genome consisting of 121,442 bp with a GC content of 37.4%, which is significantly lower than that of the host *E. coli* (about 50% GC). Genome analysis of the phage revealed 194 putative open reading frames (94.4% of the genome consists of a coding region), with 26 oriented in a forward orientation and 168 in a reverse orientation, and two tRNA genes were identified. Based on BLAST results, functions were assigned to 95 of the genes; most of the annotated genes (98 genes) were hypothetical proteins, probably due to the enormous diversity of bacteriophages and the insufficient database information about the functional genes of phage. Only one gene product is hypothetical novel proteins (Additional file 2: Table S2). The distribution of the ORFs into COG functional categories is provided in Table 4.

**Table 3:** Genome statistics

Attribute	Value	% of Total^a^
Genome size (bp)	121,442	100.00
DNA coding (bp)	114,642	94.40
DNA G + C (bp)	45,419	37.40
DNA scaffolds	1	100.00
Total genes	196	100.00
Protein coding genes	194	98.98
RNA genes	2	1.02
Pseudo genes	0	0.00
Genes in internal clusters	0	0.00
Genes with function prediction	95	48.47
Genes assigned to COGs	148	75.51
Genes with Pfam domains	62	31.96
Genes with signal peptides	5	2.57
Genes with transmembrane helices	15	7.73
CRISPR repeats	0	0.00

^a^The total is based on the total number of protein coding genes in the genome

**Table 4:** Number of genes associated with general COG functional categories

Code	Value	% of Total^a^	Description
J	5	2.55	Translation, ribosomal structure and biogenesis
A	1	0.51	RNA processing and modification
K	3	1.53	Transcription
L	17	8.67	Replication, recombination and repair
B	0	0.00	Chromatin structure and dynamics
D	2	1.02	Cell cycle control, Cell division, chromosome partitioning
V	0	0.00	Defense mechanisms
T	6	3.06	Signal transduction mechanisms
M	0	0.00	Cell wall/membrane biogenesis
N	0	0.00	Cell motility
U	0	0.00	Intracellular trafficking and secretion
O	6	3.06	Posttranslational modification, protein turnover, chaperones
C	0	0.00	Energy production and conversion
G	0	0.00	Carbohydrate transport and metabolism
E	12	6.12	Amino acid transport and metabolism
F	11	5.61	Nucleotide transport and metabolism
H	5	2.55	Coenzyme transport and metabolism
I	0	0.00	Lipid transport and metabolism
P	0	0.00	Inorganic ion transport and metabolism
Q	0	0.00	Secondary metabolites biosynthesis, transport and catabolism
R	20	10.20	General function prediction only
S	60	30.61	Function unknown
-	48	24.48	Not in COGs

^a^The total is based on the total number of protein coding genes in the genome

## Insights from the genome sequence

The results of BLAST revealed that the genome of phage phiE142 has a high similarity (query coverage, 94%; identity, 97%) with coliphage vB_EcoM_PhAPEC2, which belong to the *Tevenvirinae* subfamily of the genus T4-like viruses, an observation that is consistent with the analysis of the DNA polymerase. We therefore concluded that phiE142, based on sequence similarity, belong to the *Tevenvirinae* subfamily. However, some differences in genome organization were observed, because progressive Mauve genome alignment revealed one colinear block that is in the different order in both bacteriophages (Additional file 3: Figure S3). The principle region of genomic dissimilarity was located between 110,000 pb and 121,000 pb, this region includes a set of ORFs found to be associated with phage-host recognition, suggesting specific features of phage evolution.

The phiE142 genome is functionally organized into four modules containing gene clusters for virion morphogenesis, DNA replication/regulation, DNA packaging, and host cell lysis. This modular organization of the genome is typical of bacteriophages.

Thirty-one ORFs were found to encode proteins involved in the morphogenesis of virions. These include the ORFs 1–3, 170, 172, 175–185, and 187–194, which are proposed to be genes encoding the components of the tail fiber and baseplate. Databases homology searches suggested that ORFs encoding capsid protein are 46, 139, 142, and 174. Additionally, the proteins encoded by ORFs 185 and 186 are most similar in its amino acid sequence to neck protein.

Overall, a total of 46 ORFs are associated with processing of the viral DNA. Our analysis of the phage genomes reveals several genes potentially involved in nucleotide metabolism, including ORFs 14–15, 38–39, 47, 64, 70, 96, 100–101, 125, and 171. In addition, genes that encode proteins involved in replication and transcription of its own DNA were identified in ORFs 5, 7, 12–13, 18, 20–21, 24–25, 28–29, 32, 34–35, 37, 49, 56, 59, 61, 66, 71, 73–76, 78, 81, 86, 102, 106, 130, 132, 141, 144, and 173.

Two ORFs exhibit similarity to a gene involved in the host cell lysis, including endolysin and holin. The protein encoded by ORF 143 displays a high degree of identity with the endolysin. This ORF contained one glycohydrolase domain (hydrolyse the beta-1,4-glycosidic bond between N-acetylmuramic acid and N-acetylglucosamine), which indicates that this protein is probably an enzyme that degrades peptidoglycan. While the putative protein of ORF 4 was identified as a holin protein. Unusually, this ORF is not located adjacent to the endolysin ORF, in most genomes bacteriophages, the holin ORF is adjacent or overlaps a ORF encoding an endolysin. The deduced holin encoded by phiE142 phage has one putative transmembrane domain, and thus resembles class III holins.

The phage lifestyle prediction result of PHACTS indicated that the phiE142 is a virulent phage, consistent with the results of genomic analysis, which revealed the absence of genes associated with the establishment and maintenance of lysogenic cycle.

The DNA packaging module includes ORF 60, which encode the putative portal protein. However, it was not possible to identify the terminase subunits.

## Conclusions

Our data suggest that phiE142 is a member of T4-like virus genus of the *Myoviridae* family and the *Tevenvirinae* subfamily. Interestingly, *in silico* analyses of phiE142 genome did not exhibit homology to known virulence-associated genes, genes involved in lysogeny nor to antibiotic resistance genes or potential immunoreactive allergens. These results indicate that phage phiE142 exhibits genetics properties suitable for evaluation as a biocontrol agent.

## Additional files

Additional file 1: Table S1. Bacterial strains used in the host range spectrum of the bacteriophage phiE142. Phage was assessed for host range by spot testing. (+) indicate positive sensitivity to phage lysis, and (-) indicate negative sensitivity to phage lysis. (DOCX 41 kb)
Additional file 2: Table S2. Predicted open reading frames (ORFs) of phiE142 and predicted database matches (DOCX 60 kb)
Additional file 3: Figure S3. Comparison of genome sequence of bacteriophages phiE142 and vB_EcoM_PhAPEC2. The comparison was carried out with progressive MAUVE. (JPG 177 kb)

## Abbreviations

GOLD: Genomes On Line Database
ORFs: Open reading frames
PHACTS: Phage Classification Tool Set
TEM: Transmission electron microscopy
TSA: Tryptic soy agar
TSB: Tryptic soy broth

## References

[1] Torgerson PR, de Silva NR, Fèvre EM, Kasuga F, Rokni MB, Zhou X-N (2014). The global burden of foodborne parasitic diseases: An update. Trends Parasitol.

[2] Ahmed A, Shimamoto T (2013). Isolation and molecular characterization of *Salmonella enterica*, *Escherichia coli* O157:H7 and *Shigella* spp. from meat and dairy products in Egypt. Int J Food Microbiol.

[3] Johannessen GS, Eckner KF, Heiberg N, Monshaugen M, Begum M, Økland M (2015). Occurrence of *Escherichia coli*, *Campylobacter*, *Salmonella* and Shiga-Toxin producing *E. coli* in Norwegian primary strawberry production. Int J Environ Res Public Health.

[4] Ghasemi SM, Bouzari M, Emtiazi G (2014). Preliminary characterization of *Lactococcus garvieae* bacteriophage isolated from wastewater as a potential agent for biological control of lactococcosis in aquaculture. Aquacult Int.

[5] Carlton R, Noordman W, Biswas B, de Meester ED, Loessner M (2005). Bacteriophage P100 for control of *Listeria monocytogenes* in foods: Genome sequence, bioinformatic analyses, oral toxicity study, and application. Regul Toxicol Pharmacol.

[6] Hudson J, Billington C, Wilson T, On S (2013). Effect of phage and host concentration on the inactivation of *Escherichia coli* O157: H7 on cooked and raw beef. Food Sci Technol Int.

[7] Hagens S, Loessner MJ (2007). Application of bacteriophages for detection and control of foodborne pathogens. Appl Microbiol Biotechnol..

[8] Hungaro HM, Mendonça RCS, Gouvêa DM, Vanetti MCD, de Oliveira PCL (2013). Use of bacteriophages to reduce in chicken skin in comparison with chemical agents. Food Res Int.

[9] CastrodelCampo N, Amarillas Bueno LA, García Camarena MG, Chaidez Quiroz C, León Félix J, Martínez Rodríguez CI (2011). Presencia de *Salmonella* y *Escherichia coli* O157:H7 en la zona centro del estado de Sinaloa y su control biológico mediante el uso de bacteriófagos [abstract no. C39].

[10] Amézquita-López B, Quiñones B, Cooley M, León-Félix J, Campo C, Mandrell R (2012). Genotypic analyses of Shiga toxin-producing *Escherichia coli* O157 and non-O157 recovered from feces of domestic animals on rural farms in Mexico. PLoS One.

[11] Ackermann H-W, Clokie MRJ, Kropinski A (2009). Phage classification and characterization. Methods in Molecular Biology.

[12] King AMQ, Adams MJ, Carstens EB, Lefkowitz EJ (2012). Virus taxonomy: classification and nomenclature of viruses: ninth report of the international committee on taxonomy of viruses.

[13] Carey-Smith G, Billington C, Cornelius A, Hudson J, Heinemann J (2006). Isolation and characterization of bacteriophages infecting *Salmonella* spp. FEMS Microbiol Lett.

[14] Sambrook J, Russell DW (2001). Molecular Cloning: A laboratory manual.

[15] Delcher AL, Bratke KA, Powers EC, Salzberg SL (2007). Identifying bacterial genes and endosymbiont DNA with glimmer. Bioinformatics.

[16] Besemer J, Lomsadze A, Borodovsky M (2001). GeneMarkS: A self-training method for prediction of gene starts in microbial genomes. Implications for finding sequence motifs in regulatory regions. Nucleic Acids Res.

[17] Rombel IT, Sykes KF, Rayner S, Johnston SA (2002). ORF-FINDER: A vector for high-throughput gene identification. Gene.

[18] Quevillon E, Silventoinen V, Pillai S, Harte N, Mulder N, Apweiler R, Lopez R (2005). InterProScan: Protein domains identifier. Nucleic Acids Res.

[19] Finn RD, Bateman A, Clements J, Coggill P, Eberhardt RY, Eddy SR, Heger A, Hetherington K, Holm L, Mistry J, Sonnhammer ELL, Tate J, Punta M (2014). The Pfam protein families database. Nucleic Acids Res.

[20] Krogh A, Larsson B, von Heijne G, Sonnhammer EL (2001). Predicting transmembrane protein topology with a hidden markov model: Application to complete genomes. J Mol Biol.

[21] Laslett D, Canback B (2004). ARAGORN, a program to detect tRNA genes and tmRNA genes in nucleotide sequences. Nucleic Acids Res.

[22] Lowe TM, Eddy SR (1997). TRNAscan-sE: A program for improved detection of transfer RNA genes in Genomic sequence. Nucleic Acids Res.

[23] GenSkew – visualization of nucleotide skew in genome sequences. http://mips.gsf.de/services/analysis/genskew.

[24] Chen L, Xiong Z, Sun L, Yang J, Jin Q (2011). VFDB 2012 update: Toward the genetic diversity and molecular evolution of bacterial virulence factors. Nucleic Acids Res.

[25] Kleinheinz KA, Joensen KG, Larsen MV (2014). Applying the ResFinder and VirulenceFinder web-services for easy identification of acquired antibiotic resistance and *E. coli* virulence genes in bacteriophage and prophage nucleotide sequences. Bacteriophage.

[26] Food Allergy Research and Resource Programme (FARRP). http://www.allergenonline.com.

[27] McNair K, Bailey BA, Edwards RA (2012). PHACTS, a computational approach to classifying the lifestyle of phages. Bioinformatics.

[28] Darling AE, Mau B, Perna NT (2010). Progressive Mauve: Multiple genome alignment with gene gain, loss and rearrangement. PLoS One.

[29] Field D, Garrity G, Gray T, Morrison N, Selengut J, Sterk P (2008). The minimum information about a genome sequence (MIGS) specification. Nat Biotechnol.

[30] Ashburner M, Ball CA, Blake JA, Botstein D, Butler H, Cherry JM (2000). Gene ontology: Tool for the unification of biology. Nat Genet.

