# Supplementary material for: Complete genome sequence of new bacteriophage phiE142, which causes simultaneously lysis of multidrug-resistant Escherichia coli O157:H7 and Salmonella enterica
Source: Stand Genomic Sci. 2016 Dec 13;11:89. doi: 10.1186/s40793-016-0211-5 (PMC5154165; doi:10.1186/s40793-016-0211-5)
Supplement: Additional file 1: Table S1. — Bacterial strains used in the host range spectrum of the bacteriophage phiE142. Phage was assessed for host range by spot testing. (+) indicate positive sensitivity to phage lysis, and (-) indicate negative sensitivity to phage lysis. (DOCX 41 kb) [file 40793_2016_211_MOESM1_ESM.docx]

**Additional file 1: Table S1.** Bacterial strains used in the host range spectrum of the bacteriophage phiE142. Phage was assessed for host range by spot testing. (+) indicate positive sensitivity to phage lysis, and (-) indicate negative sensitivity to phage lysis.

| Bacterial | Strain | Bacterial lysis |
| --- | --- | --- |
| *E. coli* O157:H7 | HC14-1 | + |
| *E. coli* O157:H7 | HE7-1 | + |
| *E. coli* O157:H7 | HC14-2 | + |
| *E. coli* O157:H7 | AC6-1 | + |
| *E. coli* O157:H7 | HE10-1 | - |
| *E. coli* O157:H7 | AR7-2 | - |
| *E. coli* O157:H7 | AR17-2 | - |
| *E. coli* O157:H7 | AC6-1 | - |
| *E. coli* O157:H7 | AR15-1 | - |
| *E. coli* O157:H7 | AR17-1 | - |
| *E. coli* O157:H7 | RM8744 | + |
| *E. coli* O157:H7 | RM8753 | + |
| *E. coli* O157:H7 | RM8754 | + |
| *E. coli* O157:H7 | RM8759 | + |
| *E. coli* O157:H7 | RM8767 | + |
| *E. coli* O157:H7 | RM8768 | + |
| *E. coli* O157:H7 | RM8769 | + |
| *E. coli* O157:H7 | RM8781 | + |
| *E. coli* O157:H7 | RM8920 | + |
| *E. coli* O157:H7 | RM8921 | + |
| *E. coli* O157:H7 | RM8922 | + |
| *E. coli* O157:H7 | RM8927 | + |
| *E. coli* O157:H7 | RM8928 | - |
| *E. coli* O157:H7 | RM9450 | + |
| *E. coli* O157:H7 | RM9451 | + |
| *E. coli* O157:H7 | RM9452 | + |
| *E. coli* O157:H7 | RM9453 | + |
| *E. coli* O157:H7 | RM9455 | + |
| *E. coli* O157:H7 | RM9457 | + |
| *E. coli* O157:H7 | RM9458 | + |
| *E. coli* O157:H7 | RM9459 | + |
| *E. coli* O157:H7 | RM9462 | - |
| *E. coli* O157:H7 | RM9463 | + |
| *Salmonella* Agona | AC2-346 | - |
| *Salmonella* Agona | HD5-1 | + |
| *Salmonella* Anatum | AC2-079 | - |
| *Salmonella* Anatum | CM-50 | - |
| *Salmonella* Give | CM-31 | + |
| *Salmonella* Give | HB4-2 | - |
| *Salmonella* Give | HB4-1 | + |
| *Salmonella* Give | HB4-1 | - |
| *Salmonella* Give | HB4-3 | - |
| *Salmonella* Give | HD6-3 | - |
| *Salmonella* Luciana | AC2-240 | + |
| *Salmonella* Minnesota | AC2-070 | + |
| *Salmonella* Minnesota | CM-51 | + |
| *Salmonella* Montevideo | CM-02 | - |
| *Salmonella* Montevideo | CM-52 | - |
| *Salmonella* Montevideo | AC2-370 | - |
| *Salmonella* Montevideo | S-188 | - |
| *Salmonella* Muenster | CM-08 | - |
| *Salmonella* Muenster | AC2-366 | - |
| *Salmonella* Oranienburg | AC2-041 | - |
| *Salmonella* Oranienburg | S-13 | + |
| *Salmonella* Oranienburg | AC2-100 | - |
| *Salmonella* Oranienburg | AC2-142 | - |
| *Salmonella* Oranienburg | CM-21 | - |
| *Salmonella* Oranienburg | AC2-026 | + |
| *Salmonella* Oranienburg | HC2-2 | - |
| *Salmonella* Oranienburg | HC2-1 | - |
| *Salmonella* Oranienburg | HC2-3 | - |
| *Salmonella* Oranienburg | HD5-2 | - |
| *Salmonella* Oranienburg | HE6-1 | - |
| *Salmonella* Oranienbur*g* | S-190 | + |
| *Salmonella* Oranienburg | S-228 | - |
| *Salmonella* Oraniengurg | S-60 | + |
| *Salmonella* Pomona | AC2-248 | - |
| *Salmonella* Poona | CM-18 | + |
| *Salmonella* Poona | HE16-1 | + |
| *Salmonella* Saintpaul | AC2-046 | - |
| *Salmonella* Saintpaul | AC2-137 | - |
| *Salmonella* Saintpaul | CM-25 | - |
| *Salmonella* Saintpaul | AC2-098 | - |
| *Salmonella* Saintpaul | HE4-1 | - |
| *Salmonella* Saintpaul | HE4-3 | - |
| *Salmonella* Sandiego | HF6-3 | - |
| *Salmonella* Typhimurium | HD4-1 | + |
| *Salmonella* Weltevreden | AC2-039 | - |
| *Salmonella* Weltevreden | CM-08 | - |
| *Salmonella* Weltevreden | HD4-2 | - |
| *Salmonella* Weltevreden | HD4-3 | + |
