# Supplementary material for: Complete genome sequence of new bacteriophage phiE142, which causes simultaneously lysis of multidrug-resistant Escherichia coli O157:H7 and Salmonella enterica
Source: Stand Genomic Sci. 2016 Dec 13;11:89. doi: 10.1186/s40793-016-0211-5 (PMC5154165; doi:10.1186/s40793-016-0211-5)
Supplement: Additional file 2: Table S2. — Predicted open reading frames (ORFs) of phiE142 and predicted database matches (DOCX 60 kb) [file 40793_2016_211_MOESM2_ESM.docx]

| ORF | Start | Stop | Length (bp) | Direction | Size (aa) | MW (kDa) | pI | Homology | E- value | Identity (%) |
| --- | --- | --- | --- | --- | --- | --- | --- | --- | --- | --- |
| 1 | 8 | 559 | 552 | + | 184 | 19.72 | 5.77 | hinge connector of long tail fiber distal connector | 1.00E^-121^ | 98 |
| 2 | 568 | 3,867 | 3,300 | + | 1100 | 118.23 | 5.37 | long tail fiber distal subunit | 0 | 88 |
| 3 | 3,899 | 4,675 | 777 | + | 259 | 25.98 | 8.48 | tail fiber adhesin | 1.00E^-174^ | 97 |
| 4 | 4,706 | 5,362 | 657 | + | 219 | 25.21 | 7.7 | holin lysis mediator | 2.00E^-158^ | 99 |
| 5 | 5,363 | 5,635 | 273 | - | 91 | 10.59 | 5.42 | anti-sigma 70 protein | 4.00E^-56^ | 100 |
| 6 | 5,648 | 5,800 | 153 | - | 51 | 5.92 | 4.6 | hypothetical protein | 1.00E^-24^ | 98 |
| 7 | 5,797 | 6,075 | 279 | - | 93 | 10.83 | 4.48 | inhibitor of MrcBC restriction nuclease | 4.00E^-57^ | 100 |
| 8 | 6,065 | 6,184 | 120 | - | 40 | 4.66 | 10.2 | hypothetical protein | 2.00E^-16^ | 97 |
| 9 | 6,361 | 6,630 | 270 | - | 90 | 10.25 | 8.58 | hypothetical protein | 3.00E^-56^ | 97 |
| 10 | 6,655 | 7,116 | 462 | - | 154 | 17.68 | 4.78 | hypothetical protein | 1.00E^-107^ | 99 |
| 11 | 7,113 | 7,457 | 345 | - | 115 | 13.26 | 9.19 | hypothetical protein | 2.00E^-77^ | 100 |
| 12 | 7,468 | 8,100 | 633 | - | 211 | 23.48 | 8.63 | middle transcription regulator | 3.00E^-146^ | 100 |
| 13 | 8,289 | 9,614 | 1,326 | - | 442 | 50.37 | 8.46 | DNA topisomerase II medium subunit | 0 | 99 |
| 14 | 9,759 | 9,905 | 147 | - | 49 | 5.21 | 4.53 | acridine resistance protein | 5.00E^-23^ | 98 |
| 15 | 9,958 | 10,404 | 447 | - | 149 | 16.86 | 9.99 | nucleoid disruption protein | 1.00E^-102^ | 99 |
| 16 | 10,481 | 10,681 | 201 | - | 67 | 7.55 | 4.48 | hypothetical protein | 1.00E^-40^ | 100 |
| 17 | 10,933 | 11,196 | 264 | - | 88 | 10.08 | 5.18 | hypothetical protein | 1.00E^-55^ | 99 |
| 18 | 11,265 | 11,741 | 477 | - | 159 | 17.97 | 6.51 | DNA endonuclease IV | 7.00E^-110^ | 98 |
| 19 | 11,758 | 12,045 | 288 | - | 96 | 10.78 | 4.77 | hypothetical protein | 2.00E^-60^ | 99 |
| 20 | 12,086 | 13,012 | 927 | - | 309 | 34.94 | 5.35 | anti-prophage protein | 0 | 95 |
| 21 | 13,022 | 15,235 | 2,214 | - | 738 | 83.89 | 6.69 | rIIA protector from prophage-induced early lysis | 0 | 99 |
| 22 | 15,248 | 15,448 | 201 | - | 67 | 8.18 | 6.29 | hypothetical protein | 1.00E^-34^ | 97 |
| 23 | 15,541 | 16,641 | 1,101 | - | 367 | 41.38 | 6.28 | hypothetical protein | 0 | 99 |
| 24 | 16,684 | 17,481 | 798 | - | 266 | 31.13 | 10.09 | homing endonuclease | 5.00E^-25^ | 50 |
| 25 | 17,481 | 19,301 | 1,821 | - | 607 | 68.19 | 7.62 | topoisomerase II large subunit | 0 | 100 |
| 26 | 19,365 | 19,790 | 426 | - | 142 | 15.66 | 5.72 | hypothetical protein | 8.00E^-95^ | 100 |
| 27 | 19,793 | 19,972 | 180 | - | 60 | 6.93 | 7.69 | hypothetical protein | 2.00E^-35^ | 100 |
| 28 | 19,975 | 20,379 | 405 | - | 135 | 15.71 | 4.7 | mRNA metabolism modulator | 3.00E^-87^ | 92 |
| 29 | 20,379 | 20,606 | 228 | - | 76 | 9.02 | 4.33 | modifier of suppressor tRNAs | 3.00E^-45^ | 99 |
| 30 | 20,664 | 21,188 | 525 | - | 175 | 20.5 | 4.71 | hypothetical protein | 6.00E^-126^ | 100 |
| 31 | 21,248 | 21,667 | 420 | - | 140 | 15.32 | 6.72 | hypothetical protein | 1.00E^-87^ | 94 |
| 32 | 21,677 | 22,204 | 528 | - | 176 | 20.18 | 5.29 | transcription factor | 5.00E^-118^ | 97 |
| 33 | 22,225 | 22,719 | 495 | - | 165 | 18.41 | 4.51 | hypothetical protein | 1.00E^-115^ | 99 |
| 34 | 22,786 | 23,463 | 678 | - | 226 | 25.73 | 5.26 | exonuclease | 5.00E^-165^ | 100 |
| 35 | 23,473 | 24,786 | 1,314 | - | 438 | 49.81 | 8.3 | DNA-dependent ATPase/helicase | 0 | 99 |
| 36 | 24,783 | 25,094 | 312 | - | 104 | 12.23 | 9.56 | hypothetical protein | 4.00E^-67^ | 99 |
| 37 | 25,097 | 25,843 | 747 | - | 249 | 28.51 | 10.14 | putative anti-sigma factor | 1.00E^-177^ | 100 |
| 38 | 25,996 | 26,604 | 609 | - | 203 | 23.48 | 4.72 | ADP-ribosyltransferase | 9.00E^-145^ | 97 |
| 39 | 26,662 | 26,796 | 135 | - | 45 | 5.3 | 4.8 | ADP-ribosylase | 6.00E^-17^ | 95 |
| 40 | 26,852 | 27,016 | 165 | - | 55 | 6.31 | 9.57 | hypothetical protein | 2.00E^-22^ | 85 |
| 41 | 27,013 | 27,192 | 180 | - | 60 | 6.87 | 4.39 | hypothetical protein | 3.00E^-28^ | 85 |
| 42 | 27,194 | 27,658 | 465 | - | 155 | 17.93 | 8.5 | hypothetical protein | 3.00E^-106^ | 99 |
| 43 | 27,658 | 27,837 | 180 | - | 60 | 7.06 | 4.63 | hypothetical protein | 4.00E^-30^ | 93 |
| 44 | 27,834 | 28,073 | 240 | - | 80 | 8.57 | 4.68 | hypothetical protein | 7.00E^-24^ | 91 |
| 45 | 28,048 | 28,275 | 228 | - | 76 | 8.56 | 4.31 | hypothetical protein | 7.00E^-47^ | 99 |
| 46 | 28,377 | 28,613 | 237 | - | 79 | 8.55 | 6.23 | small outer capsid protein | 2.00E^-49^ | 99 |
| 47 | 28,655 | 29,176 | 522 | - | 174 | 20.2 | 4.65 | nucleoside triphosphate pyrophosphohydrolase | 1.00E^-124^ | 100 |
| 48 | 29,266 | 29,463 | 198 | + | 66 | 6.6 | 10.63 | hypothetical protein | 4.00E^-33^ | 97 |
| 49 | 29,460 | 30,482 | 1,023 | - | 341 | 39.79 | 9.29 | DNA primase | 0 | 99 |
| 50 | 30,522 | 30,989 | 468 | - | 156 | 17.2 | 10.33 | hypothetical protein | 1.00E^-108^ | 99 |
| 51 | 31,009 | 31,206 | 198 | - | 66 | 7.25 | 4.38 | hypothetical protein | 2.00E^-36^ | 95 |
| 52 | 31,208 | 31,858 | 651 | - | 217 | 24.89 | 4.69 | hypothetical protein | 2.00E^-154^ | 98 |
| 53 | 31,860 | 32,153 | 294 | - | 98 | 10.89 | 4.27 | spackle periplasmic protein | 9.00E^-65^ | 99 |
| 54 | 32,217 | 32,465 | 249 | - | 83 | 9.13 | 10.02 | immunity to superinfection membrane protein | 4.00E^-48^ | 100 |
| 55 | 32,527 | 32,895 | 369 | - | 123 | 13.79 | 8.47 | hypothetical protein | 9.00E^-85^ | 99 |
| 56 | 32,968 | 33,156 | 189 | - | 63 | 7.2 | 4.71 | discriminator of mRNA degradation | 4.00E^-34^ | 97 |
| 57 | 33,153 | 33,470 | 318 | - | 106 | 11.76 | 7.34 | hypothetical protein | 2.00E^-71^ | 99 |
| 58 | 33,526 | 33,900 | 375 | - | 125 | 13.97 | 8.87 | hypothetical protein | 3.00E^-85^ | 100 |
| 59 | 33,989 | 35,431 | 1,443 | - | 481 | 53.98 | 5.31 | DNA helicase | 0 | 99 |
| 60 | 35,441 | 35,782 | 342 | - | 114 | 13.16 | 4.67 | Portal protein | 1.00E^-75^ | 99 |
| 61 | 35,775 | 36,947 | 1,173 | - | 391 | 43.25 | 5.25 | RecA protein | 0 | 100 |
| 62 | 37,047 | 37,595 | 549 | - | 183 | 21.2 | 4.67 | hypothetical protein | 5.00E^-130^ | 99 |
| 63 | 37,597 | 38,496 | 900 | - | 300 | 35.01 | 4.97 | hypothetical protein | 0 | 98 |
| 64 | 38,496 | 39,212 | 717 | - | 239 | 27.24 | 5.96 | putative thymidylate synthase | 3.00E^-177^ | 99 |
| 65 | 39,255 | 39,494 | 240 | - | 80 | 9.14 | 9.84 | hypothetical protein | 1.00E^-46^ | 96 |
| 66 | 39,491 | 40,651 | 1,161 | - | 387 | 44.76 | 6.32 | putative peptidase | 0 | 99 |
| 67 | 40,709 | 41,095 | 387 | - | 129 | 14.76 | 6.09 | hypothetical protein | 2.00E^-89^ | 99 |
| 68 | 41,095 | 42,780 | 1,686 | - | 562 | 63.96 | 6.29 | hypothetical protein | 0 | 99 |
| 69 | 42,822 | 42,965 | 144 | - | 48 | 5.31 | 8.5 | hypothetical protein | 3.00E^-23^ | 100 |
| 70 | 42,962 | 43,597 | 636 | - | 212 | 22.88 | 7.14 | arabinose 5-phosphate isomerase | 1.00E^-95^ | 81 |
| 71 | 43,638 | 46,349 | 2,712 | - | 904 | 104.62 | 5.88 | DNA polymerase | 0 | 99 |
| 72 | 46,431 | 46,808 | 378 | - | 126 | 14.82 | 8.43 | hypothetical protein | 2.00E^-85^ | 100 |
| 73 | 46,802 | 47,365 | 564 | - | 188 | 21.94 | 6.29 | clamp loader small subunit | 5.00E^-133^ | 100 |
| 74 | 47,367 | 48,329 | 963 | - | 321 | 36.05 | 7.18 | clamp loader subunit | 0 | 99 |
| 75 | 48,405 | 49,091 | 687 | - | 229 | 25.11 | 4.73 | sliding clamp DNA polymerase | 1.00E^-163^ | 100 |
| 76 | 49,134 | 49,550 | 417 | - | 139 | 15.58 | 6 | RNA polymerase binding protein | 9.00E^-97^ | 99 |
| 77 | 49,563 | 49,751 | 189 | - | 63 | 7.29 | 4.96 | hypothetical protein | 2.00E^-37^ | 100 |
| 78 | 49,806 | 51,494 | 1,689 | - | 563 | 63.68 | 7.67 | endonuclease subunit | 0 | 99 |
| 79 | 51,491 | 51,730 | 240 | - | 80 | 9.56 | 4.05 | hypothetical protein | 9.00E^-50^ | 100 |
| 80 | 51,717 | 51,974 | 258 | - | 86 | 9.62 | 3.26 | hypothetical protein | 3.00E^-53^ | 98 |
| 81 | 51,971 | 52,990 | 1,020 | - | 340 | 39.47 | 4.74 | endonuclease subunit | 0 | 100 |
| 82 | 53,060 | 53,233 | 174 | - | 58 | 6.6 | 10.35 | hypothetical protein | 2.00E^-34^ | 100 |
| 83 | 53,273 | 53,479 | 207 | - | 69 | 7.83 | 10.44 | hypothetical protein | 1.00E^-42^ | 100 |
| 84 | 53,454 | 53,777 | 324 | - | 108 | 12.52 | 9.68 | hypothetical protein | 5.00E^-69^ | 99 |
| 85 | 53,779 | 53,994 | 216 | - | 72 | 8.47 | 4.05 | hypothetical protein | 1.00E^-44^ | 100 |
| 86 | 53,978 | 54,535 | 558 | - | 186 | 21.54 | 5.36 | RNA polymerase sigma factor | 2.00E^-135^ | 100 |
| 87 | 54,691 | 54,918 | 228 | - | 76 | 8.44 | 3.52 | hypothetical protein | 2.00E^-45^ | 99 |
| 88 | 54,915 | 55,247 | 333 | - | 111 | 12.81 | 10.48 | hypothetical protein | 3.00E^-73^ | 99 |
| 89 | 55,267 | 55,554 | 288 | - | 96 | 11.11 | 5.7 | hypothetical protein | 2.00E^-48^ | 97 |
| 90 | 55,618 | 55,818 | 201 | - | 67 | 7.99 | 5.82 | hypothetical protein | 8.00E^-42^ | 98 |
| 91 | 55,815 | 55,949 | 135 | - | 45 | 5.22 | 10.29 | hypothetical protein | 1.00E^-23^ | 98 |
| 92 | 55,957 | 56,250 | 294 | - | 98 | 11.88 | 10.3 | hypothetical protein | 4.00E^-63^ | 99 |
| 93 | 56,258 | 56,506 | 249 | - | 83 | 9.38 | 9.05 | hypothetical protein | 1.00E^-49^ | 96 |
| 94 | 56,503 | 56,685 | 183 | - | 61 | 6.92 | 4.94 | hypothetical protein | 3.00E^-35^ | 95 |
| 95 | 56,750 | 56,866 | 117 | + | 39 | 4.54 | 10.47 | hypothetical protein | …. | …. |
| 96 | 56,853 | 57,176 | 324 | - | 108 | 12.26 | 9.02 | putative glutaredoxin | 4.00E^-71^ | 99 |
| 97 | 57,148 | 57,459 | 312 | - | 104 | 12.07 | 3.74 | hypothetical protein | 5.00E^-70^ | 100 |
| 98 | 57,462 | 57,677 | 216 | - | 72 | 8.01 | 10.22 | hypothetical protein | 7.00E^-42^ | 99 |
| 99 | 57,694 | 57,795 | 102 | - | 34 | 3.99 | 4.37 | hypothetical protein | 8.00E^-14^ | 94 |
| 100 | 57,788 | 58,258 | 471 | - | 157 | 18.2 | 6.75 | anaerobic NTP reductase small subunit | 3.00E^-111^ | 99 |
| 101 | 58,255 | 60,072 | 1,818 | - | 606 | 68.1 | 6.93 | anaerobic NTP reductase large subunit | 0 | 99 |
| 102 | 60,069 | 60,542 | 474 | - | 158 | 18.15 | 8.32 | endonuclease VII | 5.00E^-111^ | 99 |
| 103 | 60,582 | 60,752 | 171 | - | 57 | 6.93 | 11.68 | hypothetical protein | 8.00E^-32^ | 98 |
| 104 | 60,881 | 61,237 | 357 | - | 119 | 13.53 | 3.81 | hypothetical protein | 3.00E^-75^ | 95 |
| 105 | 61,256 | 61,552 | 297 | - | 99 | 11.51 | 3.92 | hypothetical protein | 2.00E^-61^ | 94 |
| 106 | 61,549 | 61,812 | 264 | - | 88 | 10.03 | 7.41 | thioredoxin | 9.00E^-57^ | 99 |
| 107 | 61,805 | 62,218 | 414 | - | 138 | 15.76 | 8.01 | hypothetical protein | 3.00E^-93^ | 99 |
| 108 | 62,218 | 62,523 | 306 | - | 102 | 11.88 | 6.25 | hypothetical protein | 2.00E^-64^ | 95 |
| 109 | 62,523 | 63,473 | 951 | - | 317 | 36.25 | 9.97 | hypothetical protein | 0 | 97 |
| 110 | 63,536 | 64,495 | 960 | - | 320 | 36.36 | 9.7 | hypothetical protein | 0 | 95 |
| 111 | 64,551 | 65,546 | 996 | - | 332 | 37.86 | 7.85 | hypothetical protein | 0 | 99 |
| 112 | 65,609 | 66,136 | 528 | - | 176 | 20.64 | 8.71 | hypothetical protein | 1.00E^-120^ | 99 |
| 113 | 66,195 | 66,470 | 276 | - | 92 | 10.73 | 10.05 | hypothetical protein | 6.00E^-56^ | 97 |
| 114 | 66,478 | 67,458 | 981 | - | 327 | 36.49 | 5.03 | hypothetical protein | 0 | 99 |
| 115 | 67,529 | 67,747 | 219 | - | 73 | 7.91 | 11.32 | hypothetical protein | 5.00E^-42^ | 99 |
| 116 | 67,854 | 68,840 | 987 | - | 329 | 37.82 | 6.87 | hypothetical protein | 0 | 99 |
| 117 | 68,840 | 69,313 | 474 | - | 158 | 17.94 | 9.88 | hypothetical protein | 7.00E^-104^ | 96 |
| 118 | 69,323 | 69,853 | 531 | - | 177 | 19.65 | 4.56 | hypothetical protein | 6.00E^-91^ | 76 |
| 119 | 69,855 | 70,034 | 180 | - | 60 | 6.85 | 4.01 | hypothetical protein | 7.00E^-29^ | 92 |
| 120 | 70,031 | 70,141 | 111 | - | 37 | 4.18 | 8.96 | hypothetical protein | 3.00E^-14^ | 97 |
| 121 | 70,199 | 70,405 | 207 | - | 69 | 7.91 | 4.2 | hypothetical protein | 3.00E^-42^ | 99 |
| 122 | 70,413 | 70,592 | 180 | - | 60 | 6.89 | 4.53 | hypothetical protein | 2.00E^-35^ | 98 |
| 123 | 70,694 | 70,996 | 303 | - | 101 | 11.48 | 5.69 | hypothetical protein | 1.00E^-67^ | 98 |
| 124 | 71,009 | 71,221 | 213 | - | 71 | 8.33 | 10.84 | hypothetical protein | 2.00E^-42^ | 100 |
| 125 | 71,249 | 71,830 | 582 | - | 194 | 21.41 | 6.92 | thymidine kinase | 5.00E^-142^ | 100 |
| 126 | 71,840 | 72,022 | 183 | - | 61 | 7.13 | 3.95 | hypothetical protein | 7.00E^-34^ | 98 |
| 127 | 72,019 | 72,231 | 213 | - | 71 | 8.02 | 4.65 | hypothetical protein | 4.00E^-41^ | 91 |
| 128 | 72,222 | 72,434 | 213 | - | 71 | 8.74 | 9.47 | hypothetical protein | 3.00E^-35^ | 77 |
| 129 | 72,431 | 72,898 | 468 | - | 156 | 17.42 | 5.69 | hypothetical protein | 2.00E^-110^ | 99 |
| 130 | 72,891 | 73,229 | 339 | - | 113 | 12.53 | 8.3 | valyl-tRNA synthetase modifier | 2.00E^-75^ | 98 |
| 131 | 73,235 | 73,777 | 543 | - | 181 | 20.57 | 10.2 | hypothetical protein | 1.00E^-128^ | 99 |
| 132 | 73,786 | 74,244 | 459 | - | 153 | 17.63 | 9.81 | site-specific RNA endonuclease | 9.00E^-107^ | 98 |
| 133 | 74,306 | 74,521 | 216 | - | 72 | 8.51 | 4.22 | hypothetical protein | 9.00E^-45^ | 97 |
| 134 | 74,521 | 74,787 | 267 | - | 89 | 10.21 | 5.21 | hypothetical protein | 1.00E^-57^ | 99 |
| 135 | 74,777 | 75,001 | 225 | - | 75 | 8.28 | 3.68 | hypothetical protein | 1.00E^-46^ | 97 |
| 136 | 75,001 | 75,363 | 363 | - | 121 | 13.81 | 6.52 | hypothetical protein | 2.00E^-82^ | 99 |
| 137 | 75,370 | 75,678 | 309 | - | 103 | 11.9 | 9.5 | hypothetical protein | 3.00E^-70^ | 99 |
| 138 | 75,675 | 76,178 | 504 | - | 168 | 18.85 | 8.93 | hypothetical protein | 1.00E^-118^ | 97 |
| 139 | 76,393 | 76,971 | 579 | - | 193 | 21.51 | 10.17 | internal head protein | 9.00E^-135^ | 99 |
| 140 | 77,035 | 77,295 | 261 | - | 87 | 9.49 | 6.82 | hypothetical protein | 2.00E^-50^ | 92 |
| 141 | 77,302 | 77,715 | 414 | - | 138 | 16.07 | 9.92 | endonuclease | 2.00E^-97^ | 99 |
| 142 | 77,774 | 78,055 | 282 | - | 94 | 10.43 | 5.24 | putative internal head protein | 2.00E^-53^ | 90 |
| 143 | 78,052 | 78,540 | 489 | - | 163 | 18.34 | 10.22 | lysozyme murein hydrolase | 2.00E^-116^ | 99 |
| 144 | 78,575 | 79,030 | 456 | - | 152 | 17.39 | 7.14 | nudix hydrolase | 2.00E^-110^ | 99 |
| 145 | 79,023 | 79,259 | 237 | - | 79 | 9.12 | 3.93 | hypothetical protein | 4.00E^-47^ | 99 |
| 146 | 79,256 | 79,747 | 492 | - | 164 | 18.51 | 8.3 | hypothetical protein | 1.00E^-113^ | 99 |
| 147 | 79,747 | 80,433 | 687 | - | 229 | 26.36 | 6.03 | hypothetical protein | 5.00E^-169^ | 99 |
| 148 | 80,435 | 80,740 | 306 | - | 102 | 11.34 | 7.41 | hypothetical protein | 4.00E^-65^ | 95 |
| 149 | 80,811 | 81,767 | 957 | - | 319 | 37.23 | 7.71 | hypothetical protein | 0 | 98 |
| 150 | 81,796 | 82,035 | 240 | - | 80 | 9.27 | 4.84 | hypothetical protein | 4.00E^-52^ | 99 |
| 151 | 82,098 | 82,295 | 198 | - | 66 | 7.16 | 3.75 | hypothetical protein | 3.00E^-38^ | 98 |
| 152 | 82,305 | 82,577 | 273 | - | 91 | 9.97 | 4.56 | hypothetical protein | 2.00E^-60^ | 99% |
| 153 | 82,587 | 82,892 | 306 | - | 102 | 11.16 | 4.31 | hypothetical protein | 2.00E^-41^ | 100 |
| 154 | 82,991 | 83,362 | 372 | - | 124 | 14.4 | 9.39 | hypothetical protein | 1.00E^-85^ | 98% |
| 155 | 83,352 | 83,948 | 597 | - | 199 | 23.11 | 4.61 | hypothetical protein | 8.00E^-142^ | 97 |
| 156 | 83,998 | 84,591 | 594 | - | 198 | 22.05 | 8.78 | hypothetical protein | 2.00E^-136^ | 99 |
| 157 | 84,663 | 84,890 | 228 | - | 76 | 8.86 | 4.49 | hypothetical protein | 3.00E^-48^ | 99 |
| 158 | 84,949 | 85,479 | 531 | - | 177 | 19.48 | 6.03 | hypothetical protein | 3.00E^-106^ | 93 |
| 159 | 85,510 | 85,773 | 264 | - | 88 | 10.13 | 4.29 | hypothetical protein | 3.00E^-56^ | 97% |
| 160 | 86,015 | 86,578 | 564 | - | 188 | 20.8 | 9.98 | hypothetical protein | 1.00E^-130^ | 99 |
| 161 | 86,704 | 87,168 | 465 | - | 155 | 17.08 | 10.2 | hypothetical protein | 2.00E^-103^ | 99 |
| 162 | 87,557 | 87,901 | 345 | - | 115 | 14.08 | 8.21 | hypothetical protein | 1.00E^-74^ | 94 |
| 163 | 88,156 | 88,671 | 516 | - | 172 | 20.02 | 4.19 | hypothetical protein | 3.00E^-115^ | 98 |
| 164 | 88,676 | 88,966 | 291 | - | 97 | 11.32 | 4.51 | hypothetical protein | 5.00E^-60^ | 98 |
| 165 | 88,963 | 89,325 | 363 | - | 121 | 13.79 | 4.31 | hypothetical protein | 2.00E^-83^ | 99 |
| 166 | 89,326 | 89,511 | 186 | - | 62 | 6.55 | 8.54 | hypothetical protein | 3.00E^-27^ | 85 |
| 167 | 89,582 | 89,857 | 276 | - | 92 | 10.57 | 9.45 | hypothetical protein | 4.00E^-55^ | 100 |
| 168 | 89,907 | 90,218 | 312 | - | 104 | 11.55 | 9.97 | hypothetical protein | 9.00E^-65^ | 97 |
| 169 | 90,296 | 90,751 | 456 | - | 152 | 17.2 | 5 | hypothetical protein | 2.00E^-105^ | 99 |
| 170 | 90,751 | 90,981 | 231 | - | 77 | 8.4 | 4.07 | tail fiber chaperone | 5.00E^-41^ | 99 |
| 171 | 90,986 | 91,720 | 735 | - | 245 | 28.12 | 4.49 | deoxynucleoside monophosphate kinase | 2.00E^-173^ | 98 |
| 172 | 91,774 | 92,358 | 585 | - | 195 | 21.84 | 5.22 | tail completion and sheath stabilizer protein | 6.00E^-142^ | 100 |
| 173 | 92,461 | 93,282 | 822 | - | 274 | 31.3 | 10.67 | DNA end protector protein | 0 | 99 |
| 174 | 93,285 | 93,734 | 450 | - | 150 | 17.63 | 10.29 | head completion protein | 3.00E^-104^ | 99 |
| 175 | 93,796 | 94,371 | 576 | + | 192 | 22.43 | 7.31 | baseplate wedge subunit | 9.00E^-137^ | 100 |
| 176 | 94,425 | 96,104 | 1,680 | + | 560 | 61.46 | 5.1 | baseplate hub subunit and tail lysozyme/lysozyme | 0 | 99 |
| 177 | 96,150 | 96,623 | 474 | + | 158 | 17.9 | 4.47 | hypothetical protein | 7.00E^-109^ | 99 |
| 178 | 96,626 | 96,919 | 294 | + | 98 | 10.29 | 8.23 | baseplate hub needle | 2.00E^-63^ | 99 |
| 179 | 96,928 | 98,898 | 1,971 | + | 657 | 73.9 | 4.42 | baseplate wedge subunit | 0 | 99 |
| 180 | 98,895 | 101,993 | 3,099 | + | 1033 | 119.14 | 4.72 | baseplate wedge initiator | 0 | 99 |
| 181 | 101,986 | 102,990 | 1,005 | + | 335 | 38.21 | 4.36 | baseplate wedge subunit | 0 | 100 |
| 182 | 103,053 | 103,916 | 864 | + | 288 | 30.85 | 5.06 | baseplate wedge connector for long tail fiber | 0 | 99 |
| 183 | 103,916 | 105,730 | 1,815 | + | 605 | 66.16 | 4.23 | baseplate wedge subunit and tail pin | 0 | 99 |
| 184 | 105,730 | 106,389 | 660 | + | 220 | 23.73 | 4.6 | baseplate wedge subunit | 5.00E^-158^ | 99 |
| 185 | 106,386 | 107,936 | 1,551 | + | 517 | 55.11 | 5.62 | short tail fibers | 0 | 99 |
| 186 | 107,946 | 109,388 | 1,443 | + | 481 | 51.72 | 4.3 | fibritin neck whiskers | 0 | 99 |
| 187 | 109,421 | 110,434 | 1,014 | + | 338 | 37.81 | 8.13 | neck protein | 0 | 100 |
| 188 | 110,443 | 112,413 | 1,971 | + | 657 | 73.9 | 4.42 | baseplate wedge subunit | 0 | 99 |
| 189 | 112,410 | 115,508 | 3,099 | + | 1033 | 119.16 | 4.76 | baseplate wedge initiator | 0 | 99 |
| 190 | 115,501 | 116,505 | 1,005 | + | 335 | 38.21 | 4.36 | baseplate wedge subunit | 0 | 99 |
| 191 | 116,568 | 117,431 | 864 | + | 288 | 30.87 | 5.08 | baseplate wedge connector for long tail fiber | 0 | 99 |
| 192 | 117,431 | 119,245 | 1,815 | + | 605 | 66.16 | 4.23 | baseplate wedge subunit and tail pin | 0 | 99 |
| 193 | 119,245 | 119,904 | 660 | + | 220 | 23.73 | 4.6 | baseplate wedge subunit | 5.00E^-158^ | 99 |
| 194 | 119,901 | 121,430 | 1,530 | + | 510 | 54.29 | 5.62 | short tail fibers | 0 | 98 |
