# Supplementary figures and images for: Complete genome sequence of new bacteriophage phiE142, which causes simultaneously lysis of multidrug-resistant Escherichia coli O157:H7 and Salmonella enterica
Source: Stand Genomic Sci. 2016 Dec 13;11:89. doi: 10.1186/s40793-016-0211-5 (PMC5154165; doi:10.1186/s40793-016-0211-5)

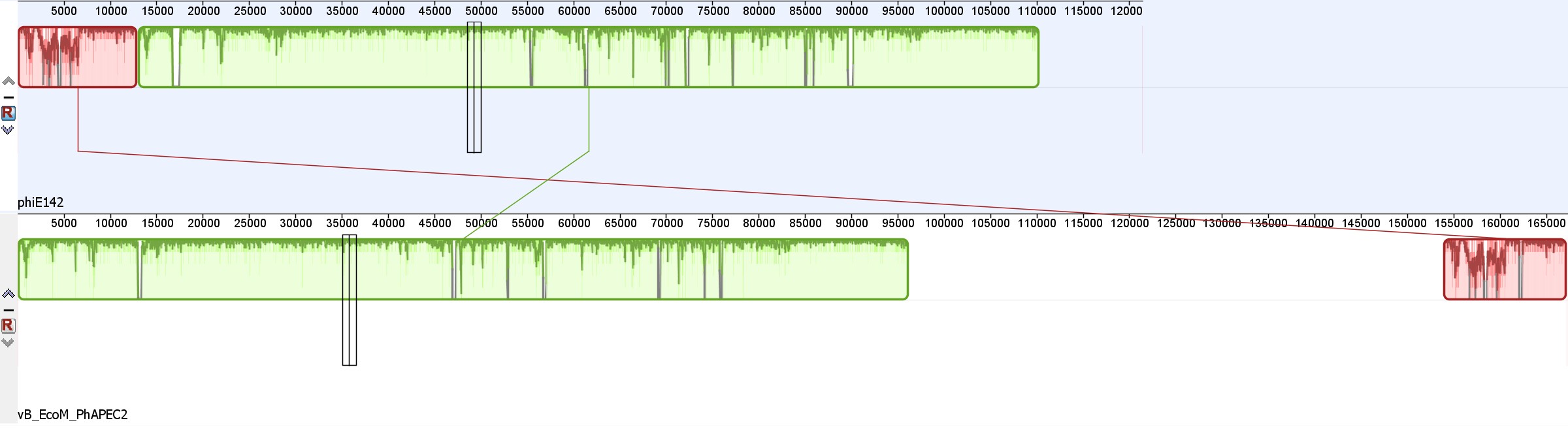

Supplement: Additional file 3: Figure S3. — Comparison of genome sequence of bacteriophages phiE142 and vB_EcoM_PhAPEC2. The comparison was carried out with progressive MAUVE. (JPG 177 kb) [file 40793_2016_211_MOESM3_ESM.jpg]
